# Caesarean Section Following Abdominoplasty With Mesh Repair: A Case Report

**DOI:** 10.7759/cureus.94215

**Published:** 2025-10-09

**Authors:** Anzara Binte Ahmed, Alina Unipan, Ratna Rani Das

**Affiliations:** 1 Obstetrics and Gynaecology, Queen’s Hospital, London, GBR; 2 Obstetrics and Gynaecology, Royal Derby Hospital, Derby, GBR; 3 Obstetrics and Gynaecology, Queen's Hospital, London, GBR

**Keywords:** abdominoplasty, caesarean section, mesh reinforcement, obstetric surgery, rectus diastasis

## Abstract

Abdominoplasty with mesh reinforcement is an increasingly used surgical technique for abdominal wall restoration, but there is limited literature regarding pregnancy and delivery following such procedures.

We report the case of a 38-year-old woman with a history of previous caesarean section and subsequent abdominoplasty with mesh repair who underwent an elective caesarean section at 39 weeks’ gestation. Intraoperatively, the mesh was encountered beneath the rectus muscles and was sharply dissected to access the peritoneal cavity. The baby was delivered via a standard lower uterine segment incision. Estimated blood loss was 1500 millilitres, and the patient had an uneventful recovery.

Literature regarding caesarean delivery after abdominoplasty with mesh is scarce, with only isolated reports available. While mesh reinforcement provides structural benefits, it raises potential concerns for subsequent pregnancy and surgical access. Our case demonstrates that caesarean section after mesh-reinforced abdominoplasty is feasible with careful surgical planning.

This report highlights the technical considerations of caesarean section in women with prior mesh repair and underscores the need for further long-term follow-up to assess maternal outcomes and abdominal wall integrity.

## Introduction

Abdominoplasty, more commonly referred to as a “tummy tuck,” is a widely performed aesthetic procedure, often in women of late reproductive age. It is also indicated for rectus diastasis, which may occur after multiple pregnancies. A variation of the procedure includes mesh reinforcement of the rectus sheath to improve abdominal wall strength and contour.

Although mesh repair is frequently used for ventral hernia surgery, its role in cosmetic abdominoplasty is less commonly reported. Given that mesh is generally recommended after childbearing, literature describing pregnancy and delivery outcomes following abdominoplasty with mesh is limited. Only a few cases of caesarean delivery after mesh reinforcement have been documented.

We present a case of successful elective caesarean section following abdominoplasty with mesh repair.

This article was previously presented as an abstract at the Royal College of Obstetrics and Gynaecology World Congress, October 2024.

## Case presentation

A 38-year-old woman, body mass index (BMI) of 39, presented at 10 weeks of gestation for antenatal booking. She had one previous caesarean section 14 years prior, complicated by chronic wound infection. Two years before conception, she underwent abdominoplasty with mesh reinforcement for both cosmetic and functional reasons.

Her antenatal course was uncomplicated. She received standard care, including serial ultrasound scans from 32 weeks, due to high BMI. The estimated fetal weight was >97th centile with normal amniotic fluid index and Doppler studies. Blood pressure remained normal, and she was at low risk for gestational diabetes.

Initially, she considered a trial of vaginal birth after caesarean section (VBAC) but opted for an elective caesarean section due to concerns of shoulder dystocia and possible intraoperative difficulties in an emergency setting. An elective caesarean section was booked at 39 weeks of pregnancy.

**Figure 1:**
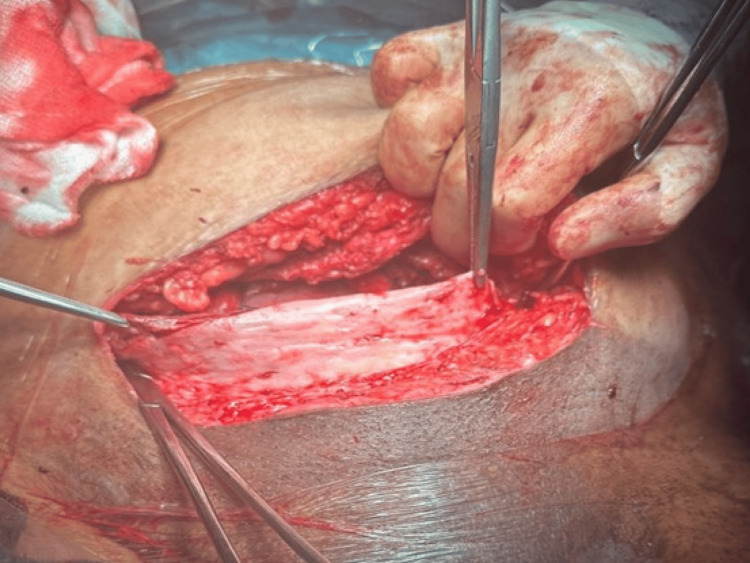
The mesh underneath the rectus sheath seen as a tough white structure

During the procedure, the abdomen was opened with a Pfannenstiel incision. Sharp dissection was done through the fat tissue, and bleeding vessels were diathermised. The rectus sheath was dissected using a scalpel. Underneath the rectus muscles, the mesh was noted as a rigid, white structure (Figure 1), which was opened with sharp dissection.

**Figure 2:**
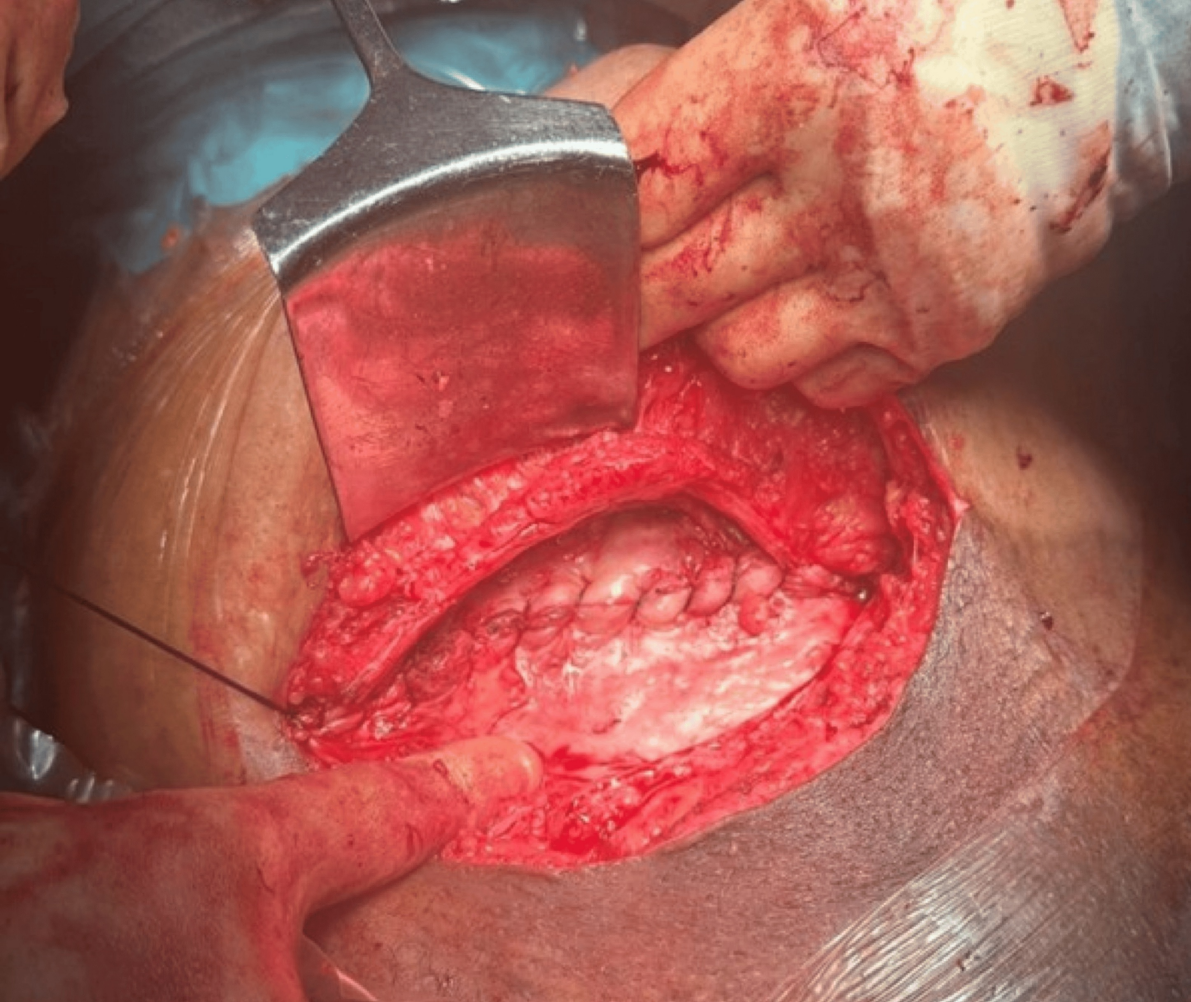
The mesh repaired with rectus sheath using PDS suture PDS: Polydioxanone

The peritoneum was opened, and the baby was delivered through a standard lower uterine incision. The placenta was delivered, and the uterus was closed in two layers. Visceral peritoneum was closed. The rectus sheath, including the mesh, was re-approximated with Polydioxanone (PDS) suture and the skin was closed with Prolene (Figure 2). Estimated blood loss was 1500ml. The patient received tranexamic acid and uterotonics during the procedure, and haemostasis was achieved. The patient had an uneventful postoperative recovery and was discharged from the hospital on the second day following caesarean section. She had a routine community midwife follow-up on the 3rd and 10th day. Prolene on the skin was removed on the 10th postoperative day. There were no concerns regarding wound healing. The patient had a further follow-up with her General Practitioner around six weeks which was unremarkable.

## Discussion

Abdominoplasty is a surgical procedure to remove excess skin and fat around the abdomen and strengthen the abdominal musculature. A classical abdominoplasty includes an extensive incision from one anterior superior iliac spine to another and around the umbilicus. A flap is created between the rectus fascia and fat, which extends superiorly up to the costal margins and xiphoid process. The rectus fascia is plicated to strengthen the muscles and narrow the waistline [1]. After careful measurements, excessive fat and skin are removed, the abdomen is closed in layers, and the umbilicus is transplanted into the flap.

There are many variations of abdominoplasty, which can be tailored to each patient's unique body type and individual needs. One such variation is mesh reinforcement. During abdominoplasty, stretched, atrophic, or torn fascia may be encountered, which is further stressed by the plication required for the procedure, making mesh reinforcement a useful adjunct [2,3]. When mesh reinforcement is performed, it strengthens the abdominal wall and increases tension. This has been associated with weight loss benefits by enhancing respiratory energy consumption and increasing intragastric pressure, thereby promoting early satiety with smaller amounts of food [4]. Recent studies also show mesh suture repair can provide favourable outcomes in terms of strength and durability, especially in cases of severe rectus diastasis [5,6]. This serves the purpose of rapid onset and permanent weight loss, complementing further body contouring.

Faessen et al. reported a case of an uneventful pregnancy one year after abdominoplasty with mesh placement, with vaginal delivery as the mode of birth [7]. Similarly, Ooi and Ngo described a successful caesarean section following abdominal mesh repair that had been performed after resection of a pregnancy-related desmoid tumour [8]. In contrast, Ozaki et al. reported mesh-related infection during pregnancy, illustrating the potential risks of such procedures in women of childbearing age (Table 1) [9].

**Table 1:** Summary of Published Literature on Abdominoplasty or Abdominal Mesh Repair and Subsequent Pregnancy Outcomes

Author (Year)	Study Type / Design	Procedure / Mesh Use	Pregnancy or Delivery Outcome	Key Findings / Discussion Points
Faessen et al. (2020) [7]	Case report	Abdominoplasty with mesh placement	Uneventful pregnancy; vaginal delivery	Demonstrated safety of pregnancy one year post-mesh placement; no mesh complications.
Ooi & Ngo (2017) [8]	Case report	Abdominal mesh repair for desmoid tumour	Caesarean section	Successful C-section after prior mesh repair; careful dissection through mesh required.
Ozaki et al. (2017) [9]	Case report	Mesh repair for incisional hernia	Pregnancy complicated by infection	Mesh-related infection during pregnancy highlights potential complications in childbearing women.
Oma et al. (2020) [10]	Nationwide cohort	Incisional hernia mesh repair in women of childbearing age	Not specific to delivery	Recommends mesh repair after last pregnancy due to risk of recurrence and complications.

Although several studies describe the risks of pregnancy following mesh repair for ventral hernia, there is limited evidence on successful caesarean delivery after such procedures. A large nationwide study suggested that mesh repair for incisional hernia should ideally be considered after the last pregnancy to minimise recurrence risk [10]. Randomised controlled trials have also explored whether combining hernia repair with abdominoplasty adds morbidity, concluding that complication rates are acceptable when performed by experienced surgeons [11].

Our case demonstrates that caesarean section can be successfully performed in the presence of prior mesh reinforcement. The main intraoperative challenge was dissecting through the mesh to reach the peritoneum. Closure of the mesh and fascia in continuity is essential for maintaining abdominal wall integrity.

However, the long-term outcomes of dissecting and re-suturing mesh during pregnancy remain unknown. Further reports and follow-up are needed to assess the durability of the repair, the risk of rectus diastasis recurrence, and implications for future caesarean deliveries.

## Conclusions

Caesarean section after abdominoplasty with mesh repair is feasible, though technically challenging. Surgeons should anticipate encountering mesh during abdominal entry and plan closure carefully to preserve abdominal wall integrity. More data are required to determine long-term maternal outcomes and establish guidelines for delivery in women with prior mesh reinforcement. What challenges a further caesarean section in such a patient will present is a matter of speculation.
